# The National Hospital—The Outlook

**Published:** 1901-12-21

**Authors:** 


### The Hospital

A Journal of

The Medical Sciences and Hospital Administration.

Vol. XXXI.—No. 795.] Edited by Sir Henry Burdett, K.C.B., and Solomon C. Smith, M.D., M.R.C.P. [December 21, 1901.

### The National Hospital—the Outlook.

At a special general meeting of the governors of the National Hospital for the Paralysed and Epileptic which was held last week, the new rules which had been prepared for the governance of the institution were submitted, and after some discussion were approved. It was also arranged—again after some discussion—that Mr. Burford Rawlings, who had been secretary for 35 years, should be granted a pension of £450 a year, and that in recognition of his past services he should be given a gratuity of one year's salary. So ends, it is to be hoped, a difficulty which has embarrassed the operations of this hospital for a long time past. The medical staff have won all along the line, and now the prosperity of the hospital depends upon what use they make of their victory. To understand fully how far this is the case, one must bear in mind that Mr. Rawlings could never have retained his position for so long had he not been supported by a not inconsiderable section of the governors, and that, so far as his policy represented ideas as to the proper scope of and aims of the hospital, those ideas still exist among people who are still actively interested in the work and progress of the National Hospital. To pension Mr. Rawlings is a great step, but it does not put his ideas on the shelf. Hence, while we feel gratified, as everyone must do, that once again peace should reign, we cannot shut our eyes to the fact that the forces which led to disruption had their origin far deeper than those questions of "seats upon the board," or of the position of the secretary-director, which are now finally disposed of. These forces, which, by the bye, do not apply to the National Hospital alone, have their root in the very different views taken by different people as to the proper uses of a hospital; by those who regard it as a place for the care and comfort of the sick, and those who regard it as a place in which disease is to be studied and the treatment of the sick is to be made better and better every year.

When the National Hospital was founded, it drew to itself a brilliant medical staff, and as a result it also drew an enormous number of patients suffering from very special and peculiar diseases. The wards and out-patient department were soon filled with strange cases, so that in a morning's work one might see more of certain diseases than some practitioners come across in a lifetime. Would the medical staff of such an institution, with such wonderful and unique opportunities, have been doing their duty had they contented themselves with merely giving to these patients the treatment mentioned in the text-books? Surely not! They felt, and they have consistently acted on the feeling, that it was their bounden duty to make the most of their position, and to do their best to discover new knowledge, and bring that knowledge to bear on the treatment of the patients. So the National Hospital became a place for research as well as for treatment, and so also it became famous—the one hospital in London which is known all over the world. But to people who take other views, all this is very shocking. They remember the early days when the hospital was truly a place for "care and comfort," and when the work of the hospital was in the hands of physicians. Between the governor who wished the patient to be nicely taken care of and the doctor who wanted to give him some bromides, there was no cause of difference; and so there was peace. But when surgery came along—surgery spelt with a very big "S" indeed—and not only surgery but surgeons, strong and self-reliant, then came also a spirit of doubt, showing itself in opposition to "the doctors." Perhaps it was not to be wondered at. People who regard a hospital from the "care and comfort" point of view are horrified when they hear of the progress which is being made; of the operations, the demonstrations, the pathological investigations, the museum, the monkeys' brains, and of the crowd of distinguished foreigners permeating the place. What, they say, has all this to do with the "care and comfort" of the sick. Here, then, is the problem which the triumphant medical staff now have to face, viz., How to reassure these doubters and to rally round the hospital once again the very various classes of supporters whose co-operation is essential to success. It is the adventurous surgery, and especially the fear lest, in a search for "records" by men each playing off his own bat, unjustifiable things may be done, that raises doubts and divides the camp; and we cannot but think that the medical staff, if they will but recognise this, may do much to set such doubts at rest. The recent vote of the governors shows that in the staff as a whole they have confidence. But in the judgment of individuals we fear they have not, and there no doubt is a strong suspicion abroad that the judgment of individuals is in certain cases allowed full scope, uncontrolled and untempered by that of the staff collectively. This is an abiding cause of distrust. But if it could be shown that treatment was never adopted in cases involving life except after consultation, and that the staff collectively were prepared to endorse all that is done, even by their most progressive colleagues; then, we think, there might be peace.

